# Rewarming rate of hypothermic neonates in a low-resource setting: a retrospective single-center study

**DOI:** 10.3389/fped.2023.1113897

**Published:** 2023-05-09

**Authors:** Elisa Rossi, Donald Micah Maziku, Dionis Erasto Leluko, Chiara Guadagno, Luca Brasili, Gaetano Azzimonti, Giovanni Putoto, Andrea Pietravalle, Francesco Cavallin, Daniele Trevisanuto

**Affiliations:** ^1^ Doctors with Africa CUAMM, Dar es Salaam, Tanzania; ^2^ Maternal and Child Department, Tosamaganga Council Designated Hospital, Ipamba, Tanzania; ^3^ Department of Research, Doctors with Africa CUAMM, Padova, Italy; ^4^ Independent Statistician, Solagna, Italy; ^5^ Department of Woman’s and Child’s Health, University Hospital of Padova, Padova, Italy

**Keywords:** rewarming, newborns, hypotermia, cerebral palsy, low-resource setting

## Abstract

**Background:**

Hypothermic neonates need to be promptly rewarmed but there is no strong evidence to support a rapid or a slow pace of rewarming. This study aimed to investigate the rewarming rate and its associations with clinical outcomes in hypothermic neonates born in a low-resource setting.

**Methods:**

This retrospective study evaluated the rewarming rate of hypothermic inborn neonates admitted to the Special Care Unit of Tosamaganga Hospital (Tanzania) in 2019–2020. The rewarming rate was calculated as the difference between the first normothermic value (36.5–37.5°C) and the admission temperature, divided by the time elapsed. Neurodevelopmental status at 1 month of age was assessed using the Hammersmith Neonatal Neurological Examination.

**Results:**

Median rewarming rate was 0.22°C/h (IQR: 0.11–0.41) in 344/382 (90%) hypothermic inborn infants, and was inversely correlated to admission temperature (correlation coefficient −0.36, *p* < 0.001). Rewarming rate was not associated with hypoglycemia (*p* = 0.16), late onset sepsis (*p* = 0.10), jaundice (*p* = 0.85), respiratory distress (*p* = 0.83), seizures (*p* = 0.34), length of hospital stay (*p* = 0.22) or mortality (*p* = 0.17). In 102/307 survivors who returned at follow-up visit at 1 month of age, rewarming rate was not associated with a potential correlate of cerebral palsy risk.

**Conclusions:**

Our findings did not show any significant association between rewarming rate and mortality, selected complications or abnormal neurologic exam suggestive of cerebral palsy. However, further prospective studies with strong methodological approach are required to provide conclusive evidence on this topic.

## Introduction

Worldwide, over 2 million neonates die every year, with the highest risk during the first 24 h of life (1). Postnatal temperature plays a crucial role in this context, and hypothermia has been recognized as an important risk factor for adverse neonatal outcomes in both high- and low-resource settings (2). In fact, hypothermia is strongly associated with neonatal mortality, with a dose-response effect which rapidly increases the risk of mortality when departing from normothermia (3).

In addition, hypothermic neonates may experience several complications such as bradycardia, tachypnea, apnea, distress, poor feeding, hypoglycemia, sepsis, and metabolic acidosis (2). The World Health Organization (WHO) has been stressing the importance of preventing thermal loss since 1993 (4), nevertheless the incidence of neonatal hypothermia remains unacceptable especially in low-resource settings (2, 5, 6). Hence, hypothermic neonates need to be promptly rewarmed and different options may be available for achieving normothermia (2).

Previous studies assessed the rewarming pace in the treatment of hypothermic neonates (7–13), but this aspect remains a matter of debate and official recommendations are still lacking (14). Slow rewarming may be preferred if considering its claimed protective role on cerebral flow and rapid cardiovascular changes (15–17). On the other hand, some reports advise that rapid rewarming may reduce the hazard associated with prolonged hypothermia (12, 18–20). In addition, anecdotal cases of complications such as hyperthermia, convulsions, and apnea were ascribed as reasons for avoiding rapid rewarming (15, 16, 21).

Hence, to date, there is no clear indication whether to prefer a rapid or a slow pace when rewarming hypothermic neonates. This study aimed to investigate the rewarming rate and its associations with clinical outcomes in hypothermic neonates born in a low-resource setting.

## Materials and Methods

### Study design and setting

This is a retrospective study on rewarming rate of hypothermic neonates admitted to the special care unit in a low-resource setting. The study was conducted at the Special Care Unit (SCU) of Tosamaganga District Hospital (Tanzania), where about 3,000 deliveries and 500 admissions occur every year. The hospital is a referral facility for a geographical area covering around 260,000 people for major obstetric emergencies. The SCU offers basic intensive care including intravenous therapies, phototherapy and oxygen therapy. Non-invasive positive-pressure support and mechanical ventilation are not available. At admission to the SCU, all neonates are screened for hypothermia, while hypoglycemia and hyperbilirubinemia are investigated based on clinical suspicion of abnormality.

Since 2019, the hospital provides follow-up for all discharged babies during their first year of life.

The study was part of a project approved by the Institutional Review Board of Tosamaganga Hospital (protocol number DOIRA/TCDH/VOL.016/5), which waived the need for written informed consent given the retrospective nature of the study and the use of anonymized data from hospital records. The research was performed in accordance with relevant guidelines and regulations.

### Patients

All neonates admitted with hypothermia to the SCU of Tosamaganga Hospital between January 1, 2019, and December 31, 2020 were evaluated for inclusion in the study. Exclusion criteria were (i) outborn neonates, (ii) being admitted to the SCU after the first day of life, and iii) missing data about body temperature at admission.

### Thermal management

At admission to SCU, neonatal axillary temperature was measured by the attending nurse using a digital thermometer (C202; Terumo, Tokyo, Japan). Severe/moderate hypothermia was defined as temperature <36°C, mild hypothermia as 36–36.4°C, normal temperature as 36.5–37.5°C and hyperthermia as >37.5°C (22). Hypothermic neonates were rewarmed in the SCU using three infant warmers and two incubators, which were used in manual mode due to the lack of temperature probes. The radiant warmer was set at 40% heater power and increased/decreased by 5% every 30 min according to measured temperature, until normothermia was reached. The incubator was manually set at 37°C (without humidity) until normothermia was reached. The rewarming rate was calculated as the difference between the first normothermic value (36.5–37.5°C) and the admission temperature, divided by the time elapsed.

### Neurological examination

The description of the neurological follow-up was reported elsewhere (23). Briefly, the neurological examination was carried out using the Hammersmith method because of the lack of expensive diagnostic equipment and personnel trained in neurological examination in the setting. We used the Hammersmith Neonatal Neurological Examination (HNNE) by Spittle et al. (24, 25), who used the percentiles of the score to define low risk (10th–90th centiles), medium risk (5th–10th or 90th–95th centiles), and high risk (<5th or >95th centiles) of neurodevelopmental impairment. Healthcare providers with 6 months of on-the-job training (CG and LB) examined the infants with the HNNE and classified them as low risk, medium risk, or high risk of neurodevelopmental impairment. At Tosamaganga Hospital, the neonatal follow-up program is scheduled at 1–3–6–9–12 months of age, but we focused on the 1-month assessment because of the high rate of loss to follow-up at later ages (22).

### Data collection

All data were retrieved from hospital records by hospital staff and were collected in an anonymized dataset. Diagnosis at admission was based on clinical examination because availability of laboratory and instrumental exams was limited. The definitions of diagnoses at admission were described elsewhere (23). Briefly, birth asphyxia was defined as 5-min Apgar Score below 7. Respiratory distress was defined as presence of signs of increased work of breathing (assessed by the Silverman Anderson Score) and/or hypoxemia with need for supplemental oxygen. Kramer's rule was used to classify the jaundice (26). Skin infection included abscess and omphalitis. Sepsis was defined as presence of clinical signs (i.e., fever, hypotonia, irritability) within (early onset) or after (late onset) the first 7 days of life. The threshold of 2.6 mmol/L blood glucose was used to define hypoglycemia.

### Statistical analysis

Continuous data were summarized as median and interquartile range (IQR), and categorical data as number and percentage. Rewarming rate was compared among subgroups of patients using Mann-Whitney test or Kruskal–Wallis test. Correlation between continuous variable was evaluated using Spearman correlation coefficient). Logistic regressions were used to evaluate the effect of rewarming rate on jaundice and respiratory distress, adjusting for clinically relevant confounders (birth weight, admission temperature, Apgar score at 5 min, meconium-stained fluid, caesarean section and birth asphyxia). Linear regression was used to evaluate the effect of rewarming rate on length of hospital stay, adjusting for clinically relevant confounders (birth weight, admission temperature, Apgar score at 5 min, meconium-stained fluid, caesarean section and birth asphyxia). Logistic regression was used to evaluate the effect of rewarming rate on mortality in neonates without possibly lethal congenital anomalies (cardiac heart disease, conjoined sibling, cranial malformation), adjusting for clinically relevant confounders (birth weight and Apgar score at 5 min). Multivariable analyses of hypoglycemia, late onset sepsis and seizures could not be performed due to the small occurrence of such events. Gestational age could not be included because it was largely missing. All tests were two-sided and a *p*-value less than 0.05 was considered statistically significant. Statistical analysis was performed using R 4.1 (R Foundation for Statistical Computing, Vienna, Austria) (27).

## Results

**Figure 1:**
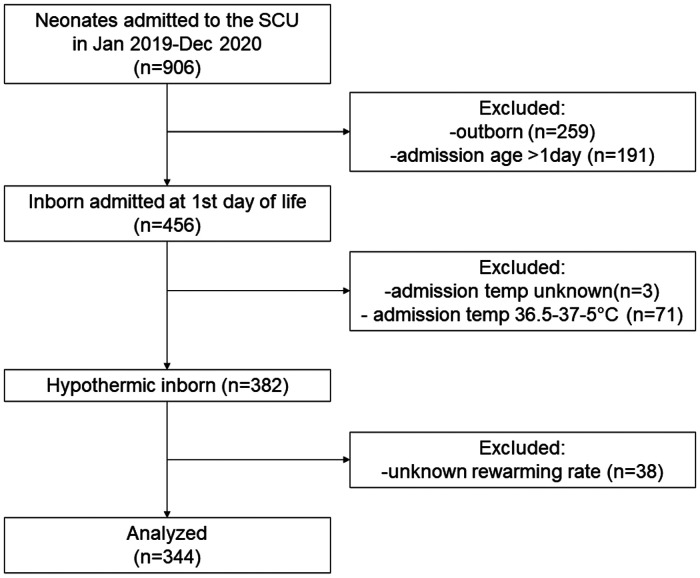
Flowchart of patient selection.

Among 906 newborn infants who were admitted to the SCU of Tosamaganga Hospital during the study period, 456 were inborn neonates admitted at the day of birth. Of them, three with unknown admission temperature and 71 normothermic infants were excluded. Among the 382 hypothermic (<36.5°C) inborn infants who were admitted at their day of birth (382/453, 84.3%), the rewarming rate could be retrieved in 344 infants (90%) who were included in the analysis (Figure 1).

Patient characteristics are reported in Table 1. Information on gestational age was largely missing (312/344, 90.7%).

**Table 1:** Patient characteristics.

N of neonates	344
Males	176 (51.2)
Birth weight, grams^a^	2,675 (1,880–3,100)
**Birth weight:**	
Normal BW (≥2,500 grams)	190 (55.2)
LBW (1,500–2,499 grams)	110 (32.0)
VLBW (1,000–1,499 grams)	38 (11.1)
ELBW (<1,000 grams)	6 (1.7)
Temperature at admission, °C^a^	35.2 (34.6–35.8)
**Temperature at admission:**	
36–36.5°C	55 (16.0)
<36°C	289 (84.0)
HIV-positive mother	30/339 (7.5)
Maternal VDRL^b^	5/331 (1.5)
Apgar score at 1 min^a^	5 (3–7)
Apgar score at 5 min^a^	7 (5–10)
PROM^c^	66 (19.2)
**Meconium:**	
Clear	243 (70.6)
Stained	101 (29.4)
Maternal fever	6 (1.7)
**Dexamethasone:**	
None	310 (90.1)
Complete cycle	22 (6.4)
Incomplete cycle	12 (3.5)
**Mode of delivery**	
Spontaneous vaginal delivery	161 (46.8)
Assisted vaginal delivery	28 (8.1)
Caesarean section	155 (45.1)
Twin pregnancy	51 (14.8)
Birth asphyxia	132 (38.4)
Respiratory distress	235 (68.3)
Early onset sepsis	18 (5.2)
Late onset sepsis	15 (4.4)
Hypoglycemia	17 (4.9)
Jaundice	77 (22.4)
Skin infection	10 (2.9)
Major malformations or chromosomopathies^d^	15 (4.4)
Seizures	37 (10.8)
Hyperthermia after rewarming	43 (12.5)

Data were summarized as *n* (%) or.

^a^ median (IQR).

^b^ VDRL was treated in 5/5 mothers.

^c^ PROM prophylaxis in 21/66 mothers.

^d^ Major malformations included cardiac heart disease (*n* = 6), club feet (*n* = 3), conjoined sibling (*n* = 2), cranial malformation (*n* = 1), Down syndrome (*n* = 1), imperforate anus (*n* = 1) and hypospadias (*n* = 1).

**Figure 2:**
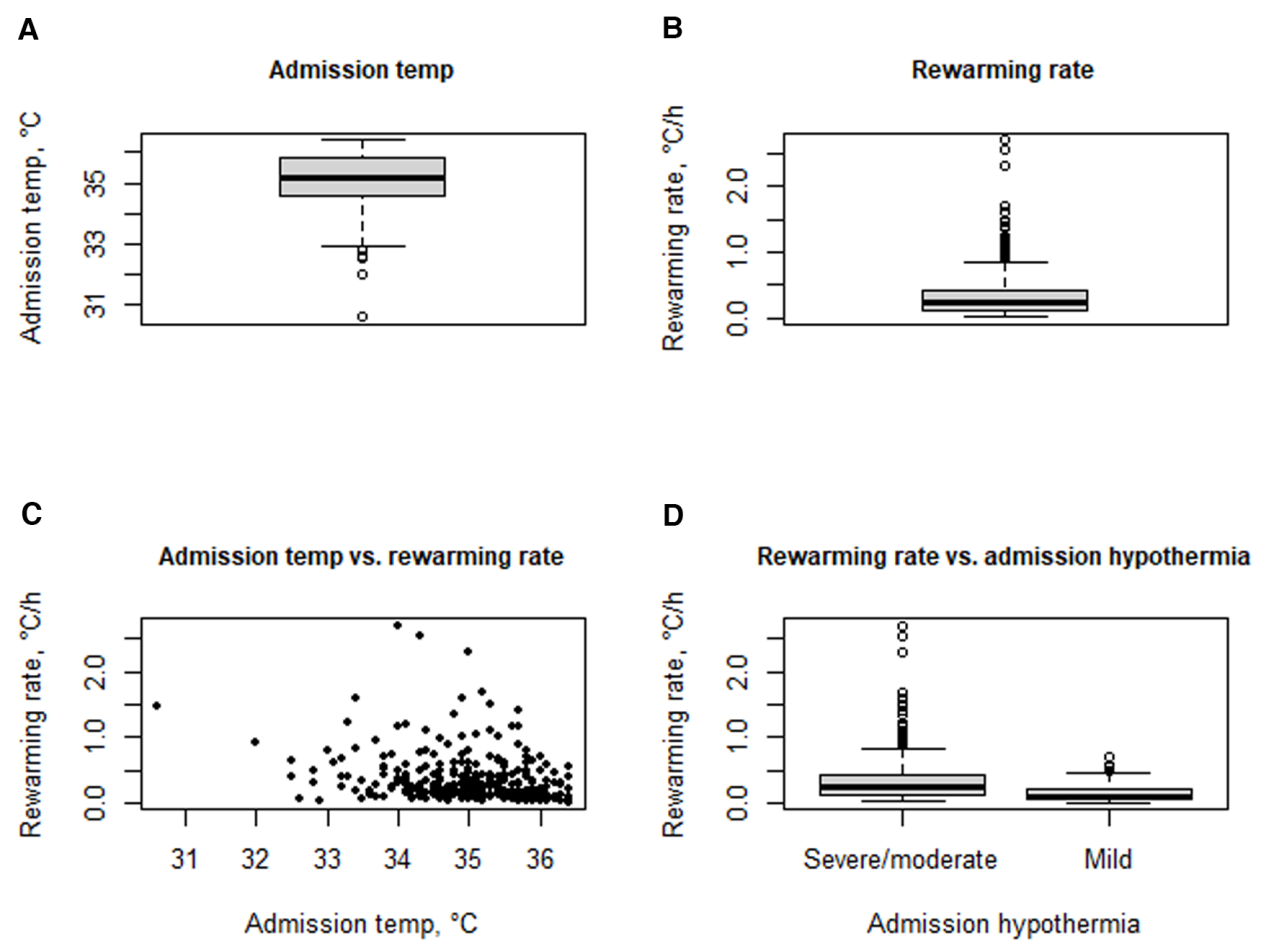
Temperature at admission (**A**); rewarming rate (**B**); correlation between admission temperature and rewarming rate (**C**); rewarming rate according to severe/moderate vs. mild hypothermia at admission.

Median temperature at admission was 35.2°C (IQR: 34.5–35.8°C; min 30.6°C, max 36.4°C) (Figure 2A). Severe/moderate hypothermia was recorded in 289 neonates (84.0%) and mild hypothermia in 55 (14.0%). Median rewarming rate was 0.22°C/h (IQR: 0.11–0.41; 0.03–2.70) (Figure 2B) and was inversely correlated to admission temperature (Spearman correlation coefficient −0.36, *p* < 0.001) (Figure 2C). Median rewarming rate was 0.24°C/h (IQR: 0.13–0.43) in neonates admitted with severe/moderate hypothermia and 0.10°C/h (IQR: 0.06–0.23) in those admitted with mild hypothermia (*p* < 0.0001 (Figure 2D). Of note, 43 neonates (12.5%) reached the hyperthermic range (>37.5°C) during the rewarming process, and they had a higher rewarming rate with respect to neonates ending up in the normothermic range (36.5–37.5°C) (*p* = 0.007, Table 2).

**Table 2:** Association between rewarming rate and clinical outcome measures.

Outcome measure	Rewarming rate was (°C/h)	*p*-value
Hypoglycemia:		0.16
No	0.22 (0.11–0.41)	
Yes	0.16 (0.07–0.22)	
Late onset sepsis:		0.10
No	0.22 (0.11–0.42)	
Yes	0.16 (0.08–0.23)	
Jaundice:		0.85
No	0.22 (0.10–0.42)	
Yes	0.20 (0.12–0.35)	
Respiratory distress:		0.83
No	0.21 (0.11–0.42)	
Yes	0.22 (0.11–0.41)	
Seizures:		0.34
No	0.22 (0.11–0.42)	
Yes	0.19 (0.08–0.30)	
Hyperthermia after rewarming:		0.007
No	0.20 (0.11–0.37)	
Yes	0.33 (0.18–0.55)	
Length of hospital stay	−0.06 ^a^	0.22

Data were summarized as median (IQR) or.

^a^ Spearman correlation coefficient.

Treatments included antibiotics (233 neonates, 67.7%), anticonvulsant (37 neonates, 10.8%) and aminophylline or caffeine (62 neonates, 18.0%). Oxygen therapy was administered to 308 neonates (89.5%) for a median of 2 days (IQR: 1–6). IV fluids were administered to 257 neonates (74.7%) for a median of 6 days (IQR: 4–9). Phototherapy was offered to 64 neonates (18.6%).

Rewarming rate was not associated with hypoglycemia (*p* = 0.16), late onset sepsis (*p* = 0.10), jaundice (*p* = 0.85), respiratory distress (*p* = 0.83), seizures (*p* = 0.34) or length of hospital stay (*p* = 0.22) (Table 2). Multivariable analyses confirmed that rewarming rate was not associated with jaundice (odds ratio 0.72, 95% confidence interval 0.36–1.90; *p* = 0.72), respiratory distress (odds ratio 0.74, 95% confidence interval 0.37–1.56; *p* = 0.41) or length of hospital stay (mean difference −2.4 days, 95% confidence interval −5.4–0.6 days; *p* = 0.11), adjusting for clinically relevant confounders. Unfortunately, multivariable analyses of hypoglycemia, late onset sepsis and seizures could not be performed due to the small occurrence of such events.

After a median length of stay of 7 days (IQR: 5–12), 37 neonates died (10.8%) while 302 were discharged (87.8%) and five were transferred to other health facilities (1.4%). Median rewarming rate was 0.26 °C/h (IQR: 0.14–0.57) in neonates who died and 0.21°C/h (IQR: 0.11–0.40) in those who did not (*p* = 0.17). At multivariable analysis, rewarming rate was not an independent predictor of mortality (odds ratio 1.27, 95% confidence interval 0.48–3.09; *p* = 0.61) in neonates without possibly lethal congenital anomalies (cardiac heart disease, conjoined sibling, cranial malformation), adjusting for clinically relevant confounders.

Later, 102 out of 307 survivors (33.2%) returned at follow-up visit at 1 month of age. The neurodevelopmental assessment suggested low potential correlate of cerebral palsy risk in 68 neonates (66.7%), moderate risk in 26 (25.5%) and high risk in 8 (7.8%). Median rewarming rate was 0.20°C/h (IQR: 0.10–0.29) in neonates with lower potential correlate of cerebral palsy risk, 0.20°C/h (IQR: 0.15–0.47) in those with moderate risk and 0.22°C/h (IQR: 0.07–0.47) in those with high risk (*p* = 0.63).

## Discussion

Our findings revealed a median rewarming rate of 0.22°C/h in hypothermic neonates, with a large variability ranging from 0.03–2.70°C/h. The rewarming rate was inversely correlated to admission temperature but was not associated with any clinical outcomes apart from the occurrence of hyperthermia with rapid rewarming.

Although avoiding heat losses immediately after birth is acknowledged as a crucial aspect in neonatal management, a substantial proportion of neonates is hypothermic at admission to intensive care unit and requires thermal intervention (2). Our data confirmed a high proportion (84.3%) of hypothermia among inborn neonates admitted to the SCU.

When dealing with cold infants, health caregivers face difficult choices as different options may be considered (using manual or automatic rewarming, setting a target temperature or a rewarming rate, using the maximum output of the warmer or adjusting the output during the process) but the optimal rewarming rate is still unknown (14). Literature offers different reasons for choosing between slow and rapid rewarming of hypothermic neonates. Some authors supported the rapid rewarming as it may lower the hazard associated with prolonged hypothermia (12, 18–20). Others argued that slow rewarming may have a protective role on cerebral flow and rapid cardiovascular changes (15–17). Of note, some authors recommended avoiding rapid rewarming on the basis of anecdotal cases of hyperthermia, convulsions or apnea (15, 16, 21). On the other hand, rapid rewarming may allow to treat a larger number of hypothermic neonates in settings with high burden of neonatal hypothermia and limited numbers of warmers machines (13). However, literature does not provide any conclusive indications whether to prefer a rapid or a slow pace when rewarming hypothermic neonates, since previous studies reported comparable clinical outcomes between the two approaches (7, 12, 13). Our data confirmed those findings, as rewarming rate was not associated with any clinical outcomes including hypoglycemia, late onset sepsis, jaundice, respiratory distress, seizures, length of hospital stay, or mortality. On the other hand, faster rewarming rate was associated with increased likelihood of reaching hyperthermia during the rewarming process, and this may exacerbate hypoxic-ischemic brain injury in asphyxiated newborns.

Of note, we did not find any association between rewarming rate and potential correlate of cerebral palsy risk at 1 month of age, but caution is suggested as only one third of the survivors attended the follow-up visit. While the HNNE has being considered a reliable assessment of neurobehaviour in the neonatal period (28), the reader should be aware that its score intervals were used as proxy for risk of cerebral palsy in our study.

Beyond the uncertainty around rewarming rate, important aspects to consider when dealing with cold infants in low-resource settings include the lack of protocol for rewarming, the lack of temperature probes to provide continuous monitoring, and the lack of skin-to-skin contact as a method for rewarming. Implementation of these approaches may contribute to reduce the burden on hypothermia but requires efforts involving organizational, cultural and economic assets.

Literature shows high heterogeneity in the rewarming rate of hypothermic neonates, with a wide range from 0.71 to 5.5°C/h (10, 13, 18, 29). Our data revealed a slower median rewarming rate (0.22°C/h), but a large range which might be due to the lack of a standardized protocol, the limited availability of warmer machines and the severity of the hypothermia. The inverse correlation between admission temperature and rewarming rate suggests that the severity of the hypothermia might have influenced the health care providers, who decided about the speed of the rewarming in absence of a standardized protocol. In our series, most hypothermic neonates were rewarmed at <0.5°C/h, which was the threshold considered in previous studies (12, 13) as it is the rewarming rate used in asphyxiated infants treated with therapeutic hypothermia (30).

The study has some limitations that should be considered when reading the results. First, the retrospective design limits the quality of the data and does not allow drawing any causal relationships, despite our results were in broad agreement with the literature. Second, the partial compliance and the short duration of the follow-up suggest caution when speculating about the long-term neurological status of the hypothermic neonates.

## Conclusions

Our findings did not show any significant association between rewarming rate and mortality, selected complications or abnormal neurologic exam suggestive of cerebral palsy. However, available information resulted from studies suffering from several limitations including the retrospective design or the small sample size, hence further prospective studies with strong methodological approach are required to provide conclusive evidence on the rewarming rate of hypothermic neonates.

## Data Availability

The raw data supporting the conclusions of this article will be made available by the authors, without undue reservation.
